# Regenerative Approach Using Intramarrow Penetration in the Management of Grade II Furcation Defect: A Case Report

**DOI:** 10.7759/cureus.98042

**Published:** 2025-11-28

**Authors:** Jignesh Tate, Saudamini More, Tejal Patil, Pranita Dalave, Prasad Mhaske

**Affiliations:** 1 Periodontology, Bharati Vidyapeeth Dental college and Hospital, Navi Mumbai, Mumbai, IND; 2 Public Health Dentistry, Bharati Vidyapeeth Dental college and Hospital, Navi Mumbai, Mumbai, IND; 3 Oral and Maxillofacial Surgery, Bharati Vidyapeeth Dental college and Hospital, Navi Mumbai, Mumbai, IND; 4 Prosthodontics, Bharati Vidyapeeth Dental College and Hospital, Navi Mumbai, Mumbai, IND

**Keywords:** alveolar bone loss, amniotic membranes, full-thickness mucoperiosteal flap, guided tissue regeneration, surgical periodontal therapy

## Abstract

Intramarrow penetration (IMP), which involves the perforation of the cortical bone to access the bone marrow, is an effective and novel approach for treating advanced furcation defects. The present case report explores the use of a regenerative technique combining IMP and guided tissue regeneration (GTR) in treating a mandibular grade II furcation defect. A 47-year-old male patient presented with pain in the lower right back region of the jaw. Initial non-surgical therapy, followed by a regenerative procedure involving IMP, bone graft placement, and resorbable membrane coverage, was employed. At six months, clinical and radiographic findings revealed successful bone regeneration, with complete defect fill and periodontal health restoration. The GTR demonstrated promising results in this patient.

## Introduction

Periodontitis is a multifactorial polymicrobial disease that leads to the destruction of the periodontal tissues, which include the gingiva, periodontal ligament, alveolar bone, and cementum. Bone loss in multirooted teeth can lead to furcation involvement because of anatomical constraints [1]. Management of furcation involvement in multirooted teeth presents a major clinical challenge in periodontology [1]. Grade II furcation defects, defined by horizontal attachment loss greater than 3 mm without complete penetration through the furcation, are particularly difficult to treat due to the anatomical complexities and limited access for instrumentation and healing [2].

The primary objective of periodontal treatment is to restore the periodontal tissues damaged by periodontitis, namely the gingiva, alveolar bone, cementum, and periodontal ligament [3]. Guided tissue regeneration (GTR) is a widely accepted approach aimed at restoring periodontal tissues by encouraging repopulation of the defect with periodontal ligament and bone cells, while preventing migration of epithelial and gingival connective tissue [4].

Although GTR shows improved outcomes in grade II furcations, complete and predictable bone regeneration remains a clinical challenge [5]. Several adjunctive strategies have been explored to enhance outcomes. One such approach is intra-marrow penetration (IMP), a method involving the perforation of the cortical bone directly inside the cancellous bone. This technique stimulates angiogenesis and enhances the recruitment of mesenchymal stem cells to the defect site, thereby improving regenerative potential [6,7].

This case report presents a well-documented and clinically significant example of a regenerative periodontal approach combining IMP and GTR with a resorbable amniotic membrane (AM) in managing a mandibular grade II furcation defect. It is relevant to clinicians and researchers interested in minimally invasive regenerative strategies and biologically driven techniques. We report a successful application of a regenerative technique using IMP along with bone graft and resorbable membrane for the management of a mandibular molar with grade II furcation involvement.

## Case presentation

Patient description and case history

A 47-year-old male reported to the Department of Periodontics with the chief complaint of pain in the lower right posterior tooth region. The patient had no known medical comorbidities. Oral examination revealed fair oral hygiene, along with localized deposits and inflammation in the mandibular posterior area. The patient also presented with a history of root canal therapy performed on the mandibular right first molar (#46) one year back.

Clinical evaluation and examination

**Figure 1:**
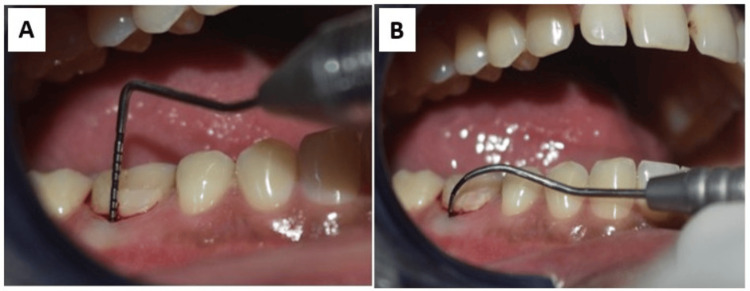
Preoperative assessment A: Pocket evaluation, B: Furcation assessment

**Figure 2:**
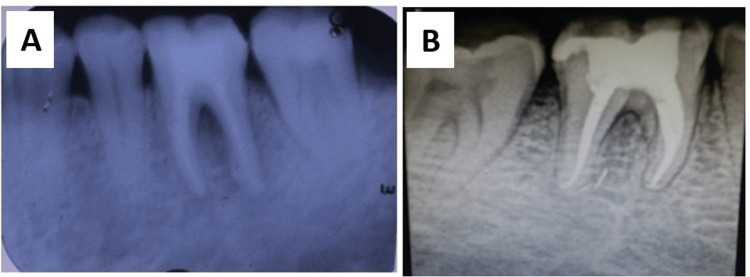
Preoperative (A) and postoperative (B) radiograph at the third month follow-up

The mandibular right first molar (#46) exhibited signs of localized chronic periodontitis. A stage III, grade B furcation defect was detected on the buccal aspect, confirmed through horizontal probing. The probing pocket depth (PPD) measured 6 mm, and clinical attachment loss was 6 mm. Tooth mobility was grade I (Figure 1). An ill-defined radiolucency was seen in the coronal and radicular aspects of #46, suggestive of furcation involvement (Figure 2, panel A).

Diagnosis and treatment

Based on clinical and radiographic findings, a diagnosis of localized chronic periodontitis with Glickman's grade II furcation involvement in tooth #46 was established. Phase I therapy (non-surgical periodontal therapy) was performed, and the patient was instructed to follow proper oral hygiene practices. The patient was reevaluated after four weeks. On clinical examination after four weeks, a residual pocket with respect to #46 was seen. Therefore, phase II therapy (surgical procedure) was planned. Under local anesthesia, a full-thickness mucoperiosteal flap was raised on both buccal and lingual aspects of #46 to provide access to the furcation defect.

Thorough debridement and root planing were carried out to remove subgingival calculus and granulation tissue. Intramarrow penetration was carried out by creating multiple small perforations in the cortical bone around the defect area using a round carbide bur under copious saline irrigation. This step aimed to induce bleeding, thereby introducing an influx of platelets and enriching the surgical site with progenitor cells and factors that help in angiogenesis (PDGF).

**Figure 3:**
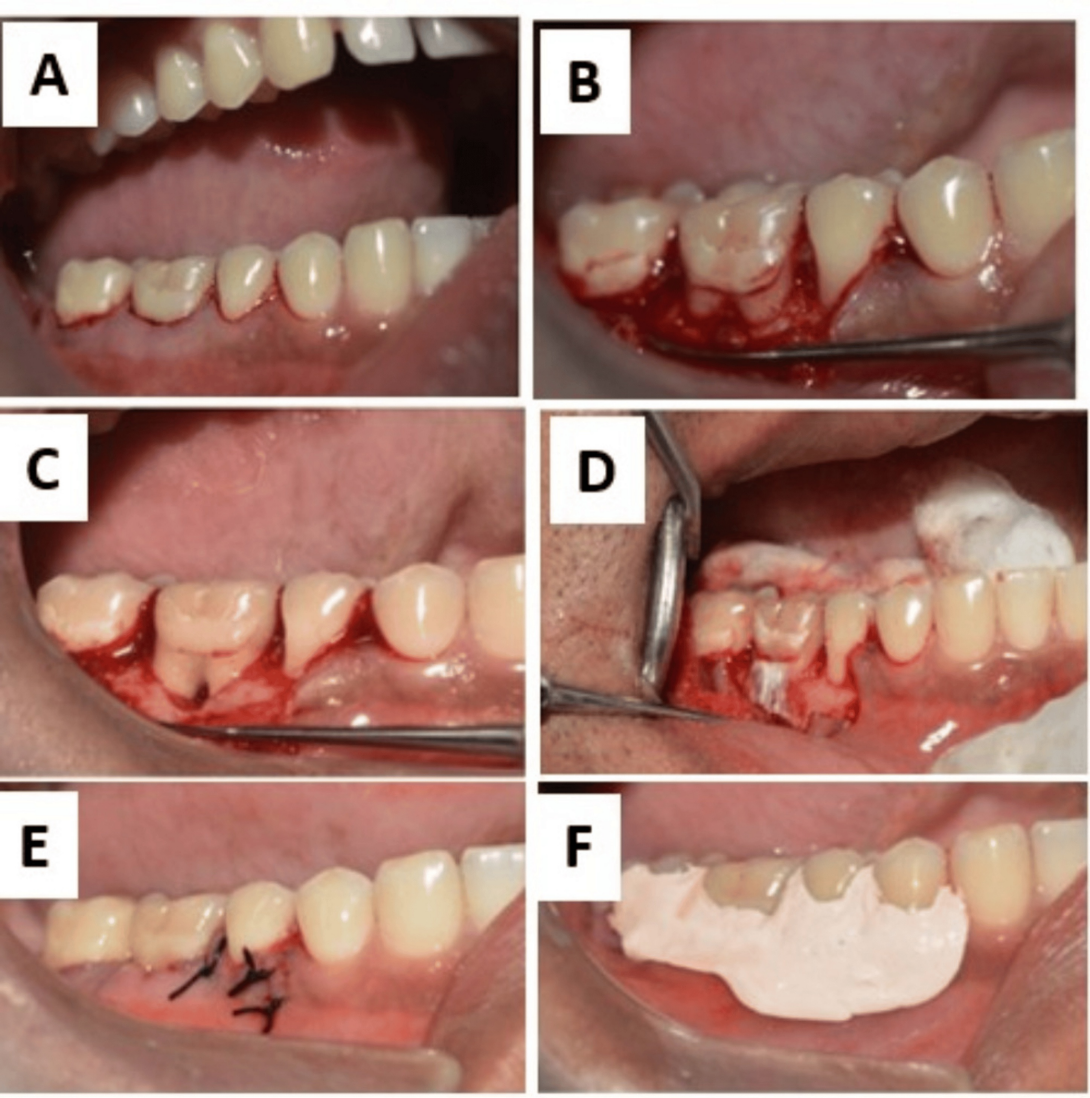
Intraoperative photographs A: Crevicular incision placed, B: Full thickness flap elevation, C: Defect debridement, D: Barrier membrane placement, E: Primary closure achieved, F: Periodontal pack placed

Following IMP, a resorbable AM was then placed over the graft (demineralized freeze-dried bone allograft (DFDBa); size: 350 x 300 microns) to serve as a barrier (Figure 3). The flap was advanced coronally, and primary closure was done using interrupted sutures, and a periodontal pack was placed over the surgical site.

Postoperative care and follow-up

The patient was prescribed an antibiotic cover of amoxicillin (500 mg) and clavulanic acid (250 mg) combination along with aceclofenac (100 mg), paracetamol (325 mg), and serratiopeptidase (15 mg) to manage inflammation. He was advised to use chlorhexidine mouthrinse (0.2%) 10 ml twice daily for 15 days. He was also instructed not to brush at the surgical site until suture removal and to avoid the consumption of spicy, hard, and hot foods.

**Figure 4:**
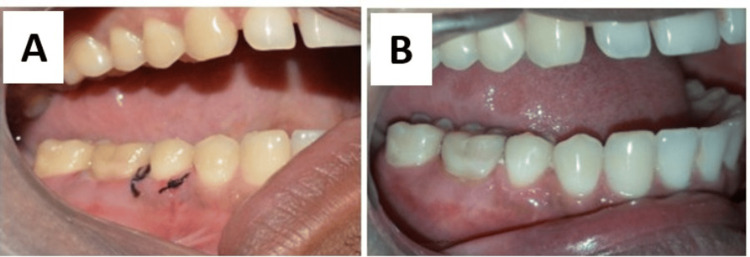
Postoperative photographs A: At the one-week follow-up, B: At the one-month follow-up

The sutures were removed after one week. Follow-up was done at one month (Figure 4) and six months after surgery. Pocket depth was assessed after one month and six months. Radiographic examination was done three months after surgery to assess bone fill (Figure 2, panel B).

Results

At the six-month follow-up, the probing pocket depth was reduced from 6 mm to 3 mm. No furcation involvement was detected upon clinical examination. Radiographs revealed significant bone fill in the furcation area. The gingival tissues were firm and healthy with no signs of inflammation or recurrence.

## Discussion

Periodontitis often leads to complex bony defects such as grade II furcation involvements, which challenge both stability and regenerative outcomes. Open flap debridement and conventional surgical therapy can arrest disease progression, but periodontal reconstruction, restoring lost periodontal ligament, cementum, and bone, is rarely achieved with predictability [8].

Guided tissue regeneration addresses this limitation by using barrier membranes to block epithelial and connective tissue migration while allowing osteoblasts and fibroblasts to repopulate. In grade II furcation, clinical trials have shown significant horizontal attachment gains and reduction in furcation involvement when GTR is employed, compared to open flap debridement alone [5,9].

Despite these advantages, GTR outcomes in furcation defects remain variable. A six-month study on mandibular grade II furcations using resorbable collagen membranes reported statistically significant gains in clinical attachment level (≈2.5 mm) and horizontal probing depth reduction (≈2.9 mm), yet none of the defects fully closed [10]. Similarly, a long-term trial over 24 months demonstrated significant horizontal attachment gain and even complete closure in select cases, but only a minority; unpredictability still lingered [5].

Laser therapy is one other recent advancement for periodontal procedures. It has proven to be more effective, particularly in achieving a reduction in probing depth and better gains in clinical attachment levels [11]. Another advancement in periodontal therapy includes 3D bioprinting. Periodontal applications of 3D printing include preparation of scaffolds, socket preservation, sinus and bone augmentation, and guided implant placement [12].

However, these advancements come with several limitations, such as high cost, limited availability, and specialized training or equipment for their use. Intramarrow penetration, also known as cortical perforation, has emerged as a useful adjunct. By creating channels into the marrow, IMP stimulates bleeding, enriches the site with progenitor cells, and improves angiogenesis, all critical to periodontal regeneration [13]. While periodontology literature on IMP specifically in furcations is limited, the biological rationale aligns well with requirements for effective GTR, especially in complex defects.

Equally promising is the use of resorbable AM as the GTR barrier. It possesses several distinct biological advantages: anti-inflammatory, antimicrobial, low immunogenicity, anti-scarring, and growth factor-rich properties that can facilitate tissue regeneration [14]. A randomized controlled trial that employed the use of the GTR technique with AM and bone grafts for intrabony defects reported greater bone fill, reduced probing depths, and improved attachment levels compared to grafts alone [15].

Recent clinical studies focus specifically on placental membranes in furcations. A clinicoradiographic study compared amnion versus chorion membranes (both with DFDBA grafts) in mandibular furcation defects, demonstrating that these membranes, enriched with inherent growth factors and antimicrobial properties, may accelerate regeneration and healing [16]. Additionally, comparisons between AM and conventional collagen membranes in intrabony defects suggest AM confers comparable or superior outcomes in probing depth reduction and attachment gain, likely due to its biologically active nature [17]. Recent systematic reviews and meta-analyses support the long-term efficacy of GTR in intrabony and furcation defects, showing significant gains in attachment level and defect fill [18,19]. However, the specific contribution of IMPs remains unclear: a recent review found only a few studies, with no direct comparisons against non-IMP protocols, although no major adverse events were reported [6]. This convergence, using GTR with resorbable AM plus IMP, combines a biologically favorable scaffold with enhanced cellular influx and vascular stimulation. In the presented case, this synergistic approach likely facilitated selective repopulation by periodontal ligament and bone cells, stabilization of the defect environment, and ultimately led to improved bone fill and clinical outcomes beyond what GTR alone might achieve.

Nevertheless, limitations include the inherent nature of a single-case report, the absence of histologic confirmation, and limited follow-up duration. Another limitation of this case report is the absence of a surgical stent or customized guiding device for standardizing probing depth and clinical attachment level measurements between the preoperative and postoperative assessments. Future randomized clinical trials with radiographic and ideally histological endpoints are essential to validate whether combining GTR, IMP, and AM consistently surpasses conventional methods in managing grade II furcation defects. Both the clinical measurements and surgical procedure were performed by the same examiner, which may introduce a potential bias in the assessment of outcomes. Key factors influencing the positive outcome in this case include proper case selection (mandibular grade II defect), good patient compliance with oral hygiene, use of biocompatible GTR membrane, surgical precision, and maintenance of a stable environment for healing.

## Conclusions

The present case highlights the successful management of a mandibular grade II furcation defect through a regenerative approach combining IMP, bone grafting, and GTR with a resorbable AM. The favorable clinical and radiographic outcomes observed at six months, including significant reduction in probing depth, gain in attachment, and complete radiographic bone fill, show the potential of this combined technique in overcoming the limitations traditionally associated with furcation therapy. The IMP enhances vascularization and progenitor cell migration, while the AM provides a biologically active scaffold with anti-inflammatory and growth factor-rich properties, creating a synergistic environment conducive to true periodontal regeneration.

This report also emphasizes the importance of proper case selection, meticulous surgical execution, and patient compliance in determining treatment success. While the encouraging results suggest that incorporating IMP with GTR may increase the predictability of furcation regeneration, the inherent limitation of a single-case report must be acknowledged. Long-term, multicenter randomized clinical trials with histological validation are needed to establish standardized protocols and confirm the reproducibility of outcomes. Nonetheless, this report adds to the growing body of evidence supporting biologically driven regenerative strategies and highlights their potential in managing complex periodontal defects.
